# The Development of Indicator Cotton Swabs for the Detection of pH in Wounds

**DOI:** 10.3390/s17061365

**Published:** 2017-06-12

**Authors:** Cindy Schaude, Eleonore Fröhlich, Claudia Meindl, Jennifer Attard, Barbara Binder, Gerhard J. Mohr

**Affiliations:** 1 JOANNEUM RESEARCH Forschungsgesellschaft mbH-Materials, Franz-Pichler-Straße 30, A-8160 Weiz, Austria; Cindy.Schaude@joanneum.at; 2 Medical University of Graz, Center for Medical Research, Stiftingtalstraße 24, A-8010 Graz, Austria; Eleonore.Froehlich@klinikum-graz.at (E.F.); Claudia.Meindl@klinikum-graz.at (C.M.); 3 Green Chemistry Centre of Excellence, University of York, York YO10 5DD, UK; ja1024@york.ac.uk; 4 Department of Dermatology and Venereology, Medical University of Graz, Auenbruggerplatz 8, A-8036 Graz, Austria; Barbara.Binder@medunigraz.at

**Keywords:** visual indicator, wound pH, pH indicator, sensor swabs, cotton swabs, traffic-light response

## Abstract

Indicator cotton swabs have been developed in order to enable faster, less expensive, and simpler information gathering of a wound status. Swabs are normally used for cleaning the wound, but here, they were covalently functionalized with indicator chemistry. Thus, they in principle enable simultaneous wound cleaning and wound pH detection. Using an indicator dye with a color change from yellow to red, combined with an inert dye of blue color, a traffic light color change from green to red is induced when pH increases. The indicator cotton swabs (ICSs) show a color change from green (appropriate wound pH) to red (elevated wound pH). This color change can be interpreted by the naked eye as well as by an optical color measurement device in order to obtain quantitative data based on the CIE L*a*b* color space. Two types of swabs have been developed—indicator cotton swabs ICS1 with a sensitive range from pH 5 to 7 and swabs ICS2 with a sensitive range from 6.5 to 8.5. The swabs are gamma-sterilized and the effect of sterilization on performance was found to be negligible. Furthermore, cytotoxicity testing shows cell viability and endotoxin levels to be within the allowable range.

## 1. Introduction

Currently, the evaluation of wounds is a qualitative rather than a quantitative issue, and accordingly, the effect of local treatment on wound healing is not well quantified. One important parameter is wound size [1], which is monitored by size scaling methods or via photography. Furthermore, coloration and wetness of wounds are described [2,3]. The intensity of pain felt by patients is another assessment method [4]. Lastly, smell may be evaluated by a nurse, but this is difficult to describe reproducibly, [5] although recent approaches attempt to make use of electronic noses [6]. Size, color, wetness, and smell are regularly reported because these parameters are easily attainable, while detection of wound colonization by specific bacteria requires much more time-consuming and costly methods such as immunoassays [7] and polymerase chain reaction [8]. Alternatively, the determination of pH in wounds may be a relevant biomarker to monitor wound healing and to foster the proper treatment of chronic wounds. If so, then the therapy can be adjusted accordingly, e.g., if the pH is too high for efficient healing, then distinct treatment to change wound pH should be applied [9]. Currently, wound pH measurement is essentially limited to electrochemical sensors [10]. The pH electrode is certainly the first choice for pH measurements, and attempts have been made to correlate wound healing with changes in wound pH [11]. Unfortunately, the pH electrode measures protons exclusively in aqueous environment and is defined for pure water containing small amounts of salts only [12]. Wound liquids do not conform to this specification, as they contain salts and biomolecules (e.g., sodium and potassium chloride, creatinine, glucose, lysocyme, matrix metalloproteinases, and proteins) in varying concentrations [13]. Another issue concerning the use of electrodes with humans is that patients do not want to have direct contact with electric current. Finally, patients do not want to be exposed to electrodes that have previously been used for other patients, even if they have been sterilized beforehand. Nevertheless, experimental data confirm that wounds do not heal properly at a pH above 8 [14,15,16].

Optical sensors have comparable limitations to electrodes in that they are, e.g., affected by ionic strength and the composition of matrix materials [17]. However, they are advantageous over electrodes with respect to their price. While it is quite expensive to discard each pH electrode after use, it is much cheaper to discard an optical sensor layer after use. This is why pH indicator paper has become so widely used [18]. It is cheap and consequently a single use item and it gives very rapid visual information, albeit of course with limited accuracy. Optical sensor layers for the detection of wound pH have already been presented [19,20] and have shown that the healing of split wounds is accompanied by a continuous decrease in pH [21]. Accordingly, while the pH of a split wound was 8.56 on the first day, the pH decreased within 14 days to a value of 6.23 [21]. The optical measurements were performed via fluorescence imaging, which gave accurate pH values over the whole area of the skin, not only at specific locations as in the case of pH electrodes. Unfortunately, the chemically instable fluorescent dyes were only sterilizable in ethanol solutions, and not by standard techniques such as gamma irradiation and ethylene oxide treatment. Furthermore, some patients may not wish to be evaluated via the camera-type devices necessary for fluorescence measurements. In contrast, they are already accustomed to being treated with cotton swabs for wound cleaning.

Based on the above considerations, we have decided to develop a simpler approach to determine wound pH. The test system is a combination of cotton swabs used for cleaning wounds and pH indicator strips used for pH measurement. In order to make this system useful for doctors and nursing personnel, it has to be cheap, the measurement must be visible and easily interpretable, and of course the material must be sterilizable and non-toxic. Accordingly, we developed pH indicator cotton swabs where the indicator chemistry is covalently immobilized to avoid contamination of wounds. Then, the effect of gamma sterilization on the response of the indicator cotton swabs was evaluated, and cytotoxicity of the swabs was tested. Finally, we performed preliminary measurements in a simulated environment, e.g., the pH of wet wound dressings and the pH of horse serum samples were measured.

## 2. Materials and Methods

### 2.1. Materials

The chemicals for coloring the cellulose-based cotton swabs and the buffers for spectral evaluation (sodium carbonate, sodium hydroxide, concentrated sulfuric acid, sodium dihydrogen phosphate, disodium hydrogen phosphate, and hydrochloric acid, all of analytical reagent grade), RINGER tablets for the preparation of RINGER’S solution, as well as disodium 1-amino-9,10-dioxo-4-[3-(2-sulfonatooxyethylsulfonyl)anilino]-anthracene-2-sulfonate, also known as Remazol Brilliant Blue R (RBBR) were from Aldrich (Vienna, Austria). The pH indicator dyes 2-fluoro-4-[4-(2-hydroxyethanesulfonyl)-phenylazo]-6-methoxyphenol (GJM-492) and 4-[4-(2-hydroxyethanesulfonyl)-phenylazo]-2,6-dimethylphenol (GJM-503) were from Joanneum Research Forschungsgesellschaft mbH (Weiz, Austria). The sterile cotton swabs (Cod.6100/SG/CS) with a length of 150 mm, a plastic shaft, and a Rayon head were from Nuova Aptaca S.R.L. (Canelli, Italy).

### 2.2. Fabrication of Indicator Cotton Swabs

In a typical immobilization procedure, each 50 mg of GJM-492 or of GJM-503 were treated with 0.5 g of concentrated sulfuric acid for 30 min at room temperature [22,23]. This converted the 2-hydroxyethylsulfonyl group of the respective indicator dye into the sulfonate. Then, the mixture was poured into 360 mL of distilled water and neutralized with 1.0 mL of a 30% sodium hydroxide solution. At this stage, 40 mg of the inert dye RBBR in 40 mL of water was added, followed by the addition of 12.5 g of sodium carbonate in 100 mL of water and 2.5 mL of a 30% sodium hydroxide solution. The cotton swabs were placed into this dyeing bath. Upon the addition of sodium hydroxide and sodium carbonate, the dye sulfonates were converted into vinylsulfonyl derivatives and coupled via Michael addition to the hydroxyl groups of the cellulose. After 30 min, the colored cotton swabs were removed from the dyeing bath and washed with copious amounts of distilled water until a green color was obtained, indicating the full removal of the alkaline reaction medium. The combination of GJM-492 with RBBR gave indicator cotton swabs type 1 (ICS1), while combination of GJM-503 with RBBR gave indicator cotton swabs type 2 (ICS2).

### 2.3. Measurements

A glass microelectrode pH meter (Hanna Instruments) was used to measure the pH of the buffered solutions. A 0.1 mol·L^−1^ phosphate buffer composed of sodium dihydrogen phosphate and disodium hydrogen phosphate was used. In order to reach pH values outside the normal buffer range, 1.0 mol·L^−1^ aqueous sodium hydroxide or 1.0 mol·L^−1^ hydrochloric acid were added. In order to represent a more realistic sample, horse serum (donor herd, USA origin, sterile-filtered, suitable for cell culture, suitable for hybridoma) from Sigma was used and was adjusted in pH using 6 N hydrochloric acid. The indicator swabs were dipped into the buffer solution for 10 s and afterwards placed under a color measurement device at 20 ± 2 °C. The color changes were given in a* values, which represent the green–red axis (negative values indicate green while positive values indicate magenta) of the L*a*b* color space, where dimension L* represents lightness, and a* and b* represent the color-opponent dimensions [24]. The pKa values were calculated by taking the a* values of the indicator cotton swabs and depicting them against pH, fitting the corresponding data with the Boltzmann fit of OriginPro 8.6 G, and calculating the points of inflection of the resulting sigmoidal graphs. The fit function was also used to calculate pH values from a* data of horse serum. Due to the smaller size of the cotton swabs relative to the illumination area of the device, black non-reflective uncoated paper was chosen as a standard color background for the cotton swabs. For experiments with wound dressings (Mepilex from Mölnlycke Health Care (Vienna, Austria), AQUACEL Extra from ConvaTec (Vienna, Austria), Suprasorb A from Lohmann & Rauscher (Vienna, Austria)), the above phosphate buffers and Ringer solution were adjusted to the appropriate pH by an addition of aqueous sodium hydroxide and hydrochloric acid.

### 2.4. Determination of Endotoxin

Detection of endotoxin was performed in compliance with ISO 10993-1 and ISO 10993-12 [25,26]. Exactly 0.2 g of the cotton swab (i.e., three pieces of cotton including the 1.5 cm long plastic stick to which the cotton is attached in 1.5 mL) were extracted per mL of pyrogen-free water for 24 ± 1 h at 37 ± 1 °C. Dilutions of this extract were also prepared with pyrogen-free water, and PYROGENT Plus 200 test (sensitivity = 0.06 EU mL^−1^, Lonza, Walkersville, MD, USA) was used for endotoxin detection. Each sample dilution was tested in duplicate, and the different endotoxin standards with *E. coli* strain 055:B5 in triplicates. One hundred microliters of standard water or samples together with 100 µL of the reconstitute lysate were added to each tube and placed at 37 °C ± 1 °C in a non-circulating water bath. After 1 h (±2 min) of incubation, each tube was examined. The reaction of each tube was recorded as either positive or negative. A positive reaction was characterized by the formation of a firm gel, which remained intact when the tube was inverted (vertical rotation of 180°). A negative reaction was characterized by the absence of a solid clot after inversion. For conversion of the qualitative data into quantification of endotoxin content, the equation supplied by the producer was used.

### 2.5. Cytotoxicity Screening of Eluates/Extracts of Cotton Swab Components

MRC-5 cells, fibroblasts derived from normal lung tissue of a 14-week-old male fetus, [27] were used for testing. Cells were cultured in 175 cm^2^ culture flasks (Costar Corning) in Minimal Essential Medium (MEM, Thermo Fisher Scientific, Vienna, Austria) + Earle’s salts, 10% fetal bovine serum (Thermo Fisher Scientific), 2 mM l-glutamine, 2% penicillin/streptomycin at 37 ± 1 °C in 5% CO_2_, and subcultured at regular intervals. Eluates from the test materials were obtained by incubation of 0.2 g·mL^−1^ of the cotton swab (i.e., five pieces of cotton including the 1 cm long plastic stick to which cotton is attached was incubated in 2.5 mL) per mL of MEM Earle’s salts, 10% fetal bovine serum (Thermo Fisher Scientific), and 2 mM l-glutamine for 24 ± 1 h at 37 ± 1 °C. The extraction was carried out in compliance with ISO 10993-5 and ISO 10993-12 [26,28]. To obtain the subconfluent cultures required for cytotoxicity testing, 16,000 MRC-5 cells were seeded per well of a 96-well plate 24 h prior to exposure to the eluates. Pure eluates and dilutions were added to the cells and exposed for 24 h. Plain polystyrene particles with a diameter of 20 nanometer (Thermo Fisher Scientific, Vienna, Austria) were used as the positive control, and plain polystyrene particles with a diameter of 200 nanometer (Thermo Scientific) as the negative control. CellTiter 96^®^ AQueous Non-Radioactive Cell Proliferation Assay (Promega, Mannheim, Germany) was used for testing. The tetrazolium compound [3-(4,5-dimethylthiazol-2-yl)-5-(3-carboxymethoxyphenyl)-2-(4-sulfophenyl)-2H-tetrazolium, inner salt; MTS] and the electron coupling reagent (phenazine methosulfate; PMS) solution supplied in the assay kit were thawed, 100 µL of the PMS solution was mixed with 2 mL of MTS solution, and 20 µL of the combined MTS/PMS solution was added to 100 µL of each well. Plates were incubated for 2 h at 37 ± 1 °C in 5% CO_2_ in a cell incubator. Absorbance was read at 490 nm on a plate reader (SPECTRA MAX plus 384, Molecular Devices, Wals-Siezenheim, Austria). In parallel, cells were viewed by brightfield microscopy to confirm the MTS data. Dehydrogenase activity was used as an indicator for cell viability and was calculated according to the following equation:

Dehydrogenase activity (%) = 100 × (A_490nm_sample − A_490nm_blank)/(A_490nm_control − A_490nm_blank). (1)

Indication for cytotoxic effect according to European and American guidelines for biological evaluation of medical devices is a dehydrogenase activity of less than 70% compared to untreated controls (solvent controls). The above absorbance measurements were not compromised by any absorbance by the indicator dyes because no leaching of the dyes into the eluates was observed.

### 2.6. Cytotoxicity Screening in Direct Contact with Tips of Cotton Swabs

MRC-5 cells were seeded at densities of 300,000/well in 6-well plates 24 h prior to the experiment to reach confluence. For the evaluation of cotton swabs in direct contact with cells, cells were exposed to two cotton swabs/well in order to cover ~1/7th the growth area [29]. A copper foil (Glas-per-Klick, Berlin, Germany) served as the positive control and PVC foil (cell culture plastic ware, GE Healthcare, Vienna, Austria) as the negative control. The controls and test samples (i.e., two pieces of cotton including the 1 cm long plastic stick to which the cotton is attached) were placed on the cell layer for 24 h at 37 ± 1 °C in 5% CO_2_. After the removal of the controls and the samples, cells were viewed in phase contrast to evaluate the cell density and morphology. Subsequently, the cells were stained with 0.2% crystal violet (Merck, Darmstadt, Germany) for 20 min at RT and washed. The cell coverage on the plate and cellular morphologies were recorded.

## 3. Results and Discussion

### 3.1. Choice of Dyes and Evaluation of the pH Indicator Cotton Swabs Using a Color Measurement Device

**Figure 1:**
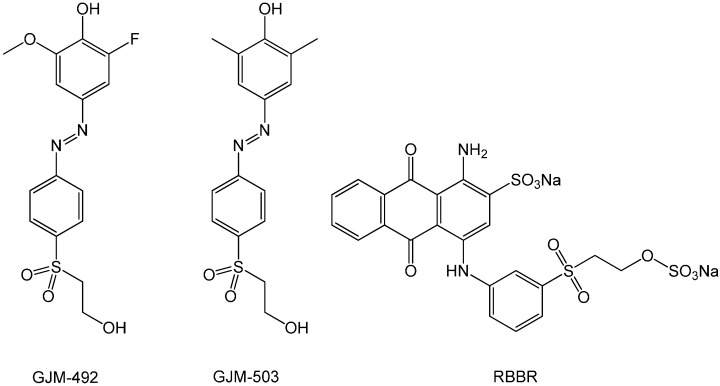
Chemical structures of the indicator dyes 2-fluoro-4-[4-(2-hydroxyethanesulfonyl)-phenylazo]-6-methoxyphenol (GJM-492) and 4-[4-(2-hydroxyethanesulfonyl)-phenylazo]-2,6-dimethylphenol (GJM-503), respectively, and the inert blue dye, Remazol Brilliant Blue R (RBBR).

**Figure 2:**
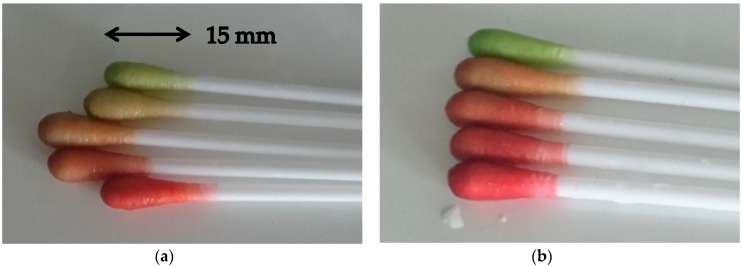
Color changes of indicator cotton swabs type 1 (ICS1) (**a**) and indicator cotton swabs type 2 (ICS2) (**b**) upon exposure to different pH buffers.

Two types of indicator swabs have been prepared; the first type made from indicator dye GJM-492 and inert dye RBBR (termed ICS1) and the second type made from indicator dye GJM-503 and inert dye RBBR (termed ICS2) (Figure 1). The indicator dyes GJM-492 and GJM-503 were chosen for their pKa values when immobilized to transparent cellulose layers, which were found to be 6.1 and 7.7, respectively [30]. These values appear to be appropriate for pH measurements in wounds, considering that the sensitive range of the dyes covers 1.5 pH units above and below the pKa. Furthermore, both dyes show color changes from yellow to red when going from acidic to alkaline pH. This color change is not that easily discernible by the human eye. However, the addition of a blue pH-insensitive dye (RBBR) in the dyeing process converts the originally yellow-to-red color change of both GJM-492 and GJM-503 into a more logical green-to-red color change. Consequently, the two types of indicator swabs can both be evaluated visually through their color change from green to red as pH increases. For this evaluation, the indicator swabs have been placed in phosphate buffer solutions of different pH and color changes were recorded via digital photography (Figure 2).

**Figure 3:**
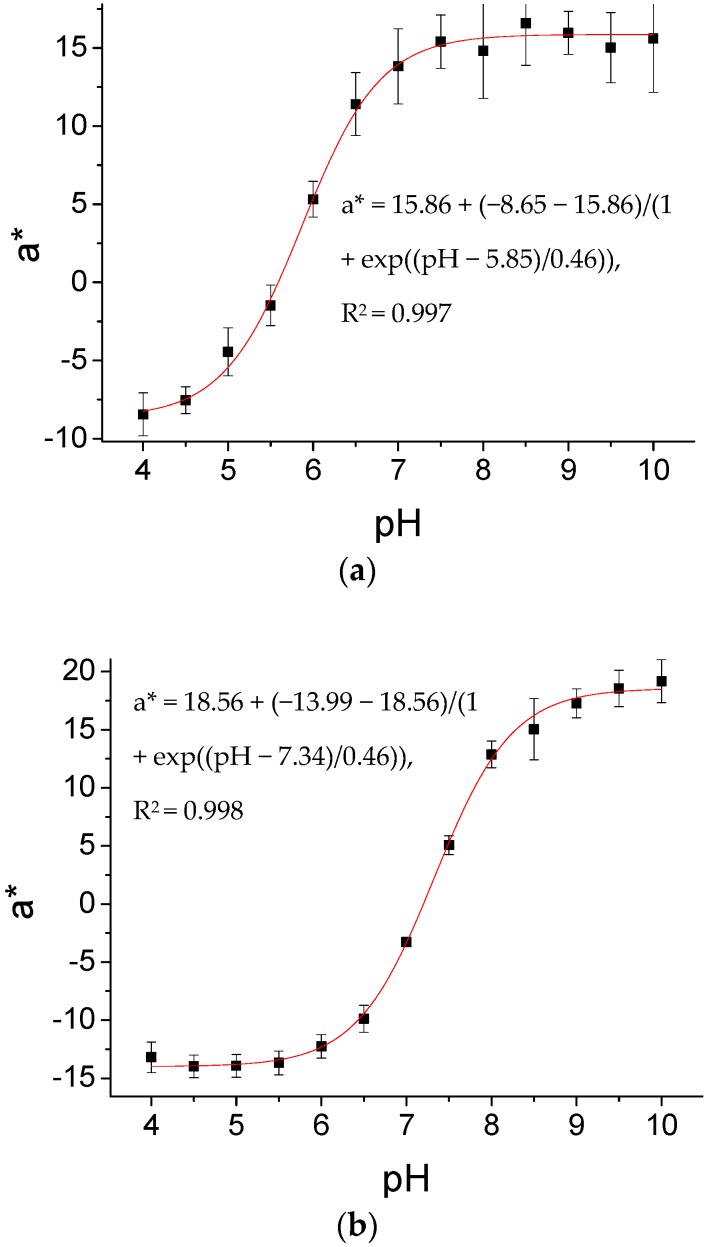
Calibration graphs of sterilized ICS1 (**a**) and ICS2 (**b**) upon exposure to different pH buffers. Five different swabs were used for error bar calculation of a* values (see also Table 1).

Visual evaluation gives rather crude information on the actual pH, because essentially three different colors—green, orange, and red—can be visualized within three pH values. However, in order to evaluate the color changes in a more quantitative way, and at the same time provide a convenient measurement method, a hand-held color measurement device was used for quantitative optical evaluation. The color measurement device gives readings of L*, a*, and b* values, which are more reliable than the RGB values of smartphones. In preliminary studies using the two different types of indicator swabs, it became clear that L* and b* values did not give reliable readings for evaluation, because L* reported luminosity/brightness rather than color changes and b* (blue to yellow) did not cover the color changes from green to red. However, a* (green to red) gave reliable readings for all pH values and thus was used for the correlation between measured color changes and pH (Figure 3). As the indicator swabs (approx. 14 × 3 − 5 mm) were smaller than the illumination area of the measurement devices (diameter of 15 mm), a black color background was used for measurements. Tests with a white background showed a signal magnitude for a* that was half of the signal magnitude with a black background. To obtain a measure for the sensitive range, pKa calculations from the readings were performed using a Boltzmann fit.

### 3.2. Sensitivities of ICS1 and ICS2, and the Effect of Sterilization

Both types of indicator cotton swabs were exposed to pH buffer solutions; afterwards, their a* values were detected using the color measurement device. Characterization of the swabs was performed before and after sterilization using 25 kGy gamma irradiation. This was done in order to evaluate any possible effect of sterilization on the performance of the indicator cotton swabs, as sterilization is well known to compromise the performance of sensing materials [31]. The pKa values for the non-sterilized indicator swabs ICS1 and ICS2 are given in Table 1. Furthermore, the table also gives data on the sterilized cotton swabs, showing that using 25 kGy gamma irradiation does not affect the indicator performance. Both the non-sterilized and sterilized indicator swabs have comparable pKa values. Thus, it can be concluded that the indicator dyes function properly before and after the sterilization process. Furthermore, the standard deviation in pKa of five different indicator swabs in comparison to measuring the same indicator swab five times is acceptable. There is a notable shift of pKa between the indicator dyes measured on transparent cellulose foils (GJM-492: 6.1, GJM-503: 7.7) and the indicator cotton swabs. This we attribute to the higher content of dye on the swabs, a concentration effect that has already been observed to shift the pKa of pH indicator dyes significantly [32,33].

### 3.3. Temperature Effect on Sensitivity

A relevant issue for optical sensors in general is the cross-sensitivity to temperature changes. Therefore, we have evaluated the indicator cotton swabs at three different temperatures; 20, 30, and 40 °C. Table 2 shows that there is a small yet significant increase in pKa with temperature. This contrasts with findings using the commonly used triphenylmethane dye phenol red, where the pKa decreases by 0.1 pH units when the temperature is raised from 25 to 37 °C [34]. We currently do not have an explanation for this finding, but the effect in both cases is approximately 0.1 pH units, which, for this application, is a minor effect, although not to be ignored.

### 3.4. Toxicity Testing of the Cotton Swabs According to ISO Guidelines

Medical devices should not cause adverse effects in the human body. Regulatory bodies like the American Society for Testing and Materials (ASTM), the Food and Drug Administration (FDA), and the International Standards Organization (ISO) provided guidelines for the testing. ISO 10993 specifies the procedure for cytotoxicity testing in eluates and in direct contact with the sample. Contamination with bacteria and components of the bacterial wall (endotoxin) in the samples has to be excluded as they are pyrogenic. The pyrogenic activity of endotoxin is much higher than that of most other pyrogenic substances. The Limulus Amebocyte Lysate (LAL) assay is an established alternative to the detection of the pyrogenic effect of endotoxin in white rabbits and is recommended by the European Medicines Agency to evaluate medical devices for bacterial endotoxins [35]. Endotoxin in the aqueous extract of the sample activates coagulase in the blood cells (amoebocytes) of the horseshoe crab [36]. The activated enzyme (coagulase) hydrolyzes specific bonds within a clotting protein (coagulogen) also present in Limulus Amebocyte Lysate. Once hydrolyzed, the resultant coagulin self-associates and forms a gelatinous clot. The LAL clotting test can be used for all types of samples. The initial rate of activation is determined by the concentration of endotoxin. The United States Pharmacopeia pyrogen standard for medical devices that contact the blood or lymph in circulation requires <20 endotoxin units (EU)/device or <5 EU/mL [37]).

In order to provide realistic testing conditions for the indicator cotton swabs, not only the cotton itself but also the adjacent plastic stick was evaluated, as this part might also come into contact with the wound during manipulation. Extracts of the originally packaged sterile uncolored cotton swaps did not induce clotting of amoebocyte lysate. This indicates endotoxin levels below the detection threshold of the assay (0.06 EU/mL). For comparison, originally uncolored sterile cotton swabs were removed from the original package and subsequently packaged again and gamma sterilized (termed manipulated swabs). Contamination with endotoxin occurred during manipulation of the cotton swabs and this could not be removed by gamma irradiation. Decontamination of samples is a well-known problem because it is very difficult to remove endotoxin from samples [38]. Extracts of the pH-sensitive sterilized cotton swabs ICS1 and ICS2 diluted to 1:5 also did not induce clotting. According to this testing, the indicator cotton swabs ICS1 and ICS2 exhibited <5 EU/mL, which is below the threshold for devices in contact with blood or lymph in circulation. Since endotoxin levels in all investigated samples (original sterile cotton swabs, cotton swabs removed from package and sterilized again, and sterilized ICS1 and ICS2) were below the accepted threshold, additional measures to reduce endotoxin contamination are not required.

As in the second step, in vitro cytotoxicity screening is recommended for identification of adverse cellular effects. Cell damage and cell death induced by the leakage of toxins can lead to an inflammatory response with recruitment of various cell types to the wound. Continuous or prolonged leakage of toxic substances will delay wound healing. Cytotoxicity testing on cell lines shows, in many cases, good correlation with animal assays. It is frequently more sensitive than animal studies and equally predictive for acute toxicity in humans to rodent in vivo studies [39]. The ISO 10993-5 guideline for cytotoxicity testing indicates evaluation of eluates and in direct contact. Total amount of proteins, DNA, cell number, or enzymatic activity of a cell population can serve as readout parameters for cell viability. The use of bioreduction of a tetrazolium dye into formazan for the assessment of cell viability is a generally accepted technique [40]. Detection with CellTiter 96^®^ AQueous Non-Radioactive Cell Proliferation Assay uses a novel tetrazolium compound MTS and the electron coupling reagent PMS. MTS is bioreduced by cellular dehydrogenases into a formazan product, which is soluble in the tissue culture medium and can be quantified by photometric measurement.

**Figure 4:**
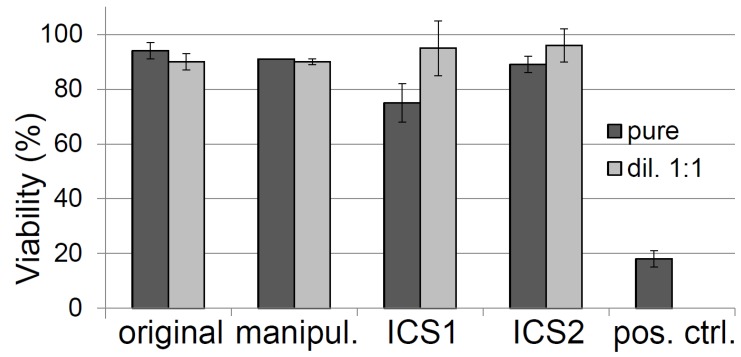
Changes in cell viality after exposure to positive control and eluates of samples (pure and diluted 1 + 1) for 24 h.

Eluate testing was performed with the original sterile uncolored cotton swabs, with the original cotton swabs removed from package and sterilized again via gamma irradiation, and with ICS1 and ICS2 after gamma sterilization. Significant decreases in viability were only observed after exposure of MRC-5 cells to the positive control and to ICS1 (Figure 4). This decrease to 75 ± 7% of the untreated cells, however, is not interpreted as cytotoxic according to ISO 10993-5, as only decreases in dehydrogenase activity below 70% are considered so [28].

**Figure 5:**
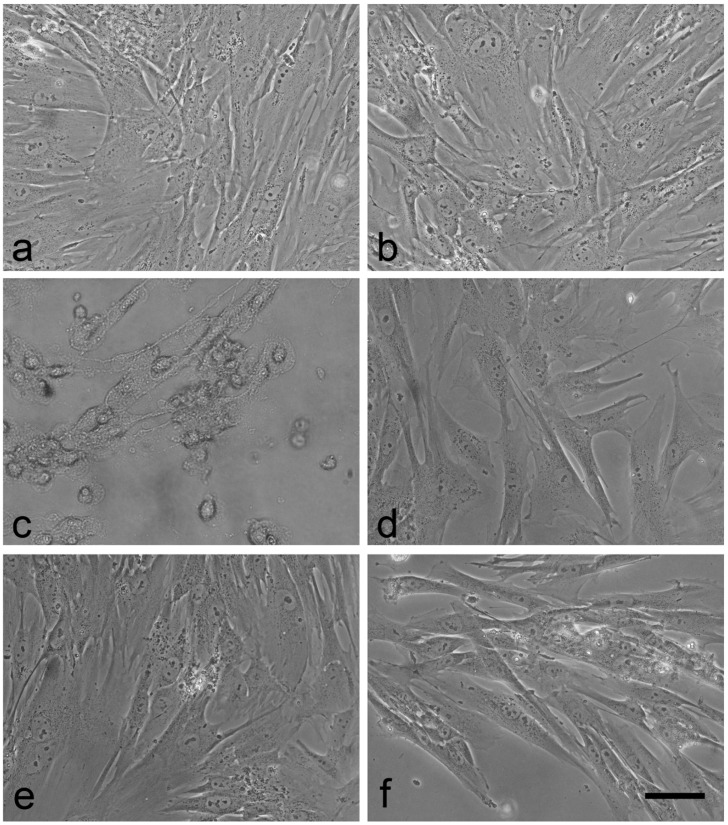
Images of untreated controls (**a**) and cells positioned in the vicinity of the samples: negative control (**b**), positive control (**c**), manipulated cotton swab (**d**), ICS1 (**e**), and ICS2 (**f**). Healthy MRC-5 cells show the elongated form of normal fibroblasts. Upon damage they round up and eventually detach from the plastic surface (**c**). Scale bar: 50 µm.

Finally, evaluation of the effects after direct contact between cells and biomedical devices identifies the presence of potentially leachable toxic materials. Damage of the cell layer is detected by colorimetric dyes and cell morphology. Cell detachment from the plate is recorded after 24 h of exposure. A confluent cell layer was observed in cultures of untreated cells and of cells exposed to repackaged and gamma irradiated (manipulated) cotton swaps, and to ICS1 and ICS2 (Figure 5a,d,e,f). On the other hand, cell density was much lower in cell cultures exposed to the positive control (Figure 5 c). Cells of the positive control were rounded and detached from the plastic support. The absence of abnormal cell morphology after direct contact with ICS1 (no indication of cell detachment, rounded cells, necrosis, etc.) related to experiments with the eluate (see above) suggests the absence of cytotoxic effects for ICS1. Decreases of dehydrogenase activity can be caused by several mechanisms and are not specific to cytotoxicity [41]. Potential reasons for reduced dehydrogenase activity are inhibition of proliferation, induction of apoptosis, and interference with mitochondrial metabolism. Additional studies would be required to identify the underlying mechanism.

### 3.5. Testing of Wound Dressings and Horse Serum Using ICS1 and ICS2

Initially, indirect evaluation of wounds was considered in an attempt to avoid contact of wounds with the indicator cotton swabs. Hence, it was decided that the pH in the wet wound dressings would be measured rather than in the wound itself. Before starting such a procedure on patients, several functional wound dressings were assessed for their effect on the pH of (a) phosphate buffer solutions and (b) Ringer solutions to confirm that dressings would not affect wound pH. The pH of phosphate buffer and Ringer solutions was measured before soaking different wound dressings. After soaking the wound dressing with the solutions, the pH in the wound dressing was measured by pressing a pH electrode into the wet dressing (Table 3).

Not surprisingly, there was a significant effect of the composition of the wound dressing on the pH inside the wound dressing. It is thought that adjusting pH in the wound establishes an environment appropriate for improved wound healing [42]. Accordingly, Mepilex seems to establish a slightly alkaline pH in phosphate buffer and in Ringer solution (6.6–8.2). Presumably, the data for the Ringer solution (7.1–7.7) is more meaningful because the wound will not have the buffer capacity of a phosphate buffer. AQUACEL and Suprasorb A seem to establish a slightly acidic environment in the Ringer solution (around 5.0–5.6). As a consequence, this preliminary experiment indicates that it is not justified to draw conclusions about the pH inside the actual wound from the pH in the wet dressing. Therefore, rather than measuring the pH indirectly, a pH measurement has to be performed directly in the wound, after removal of the functional dressing.

We performed additional experiments with horse serum to provide more realistic samples for pH measurements. The sigmoidal calibration function was calculated using phosphate buffered solutions of different pH (Figure 3). Then, a*-values for horse serum, adjusted to different pH levels by the addition of 6 N hydrochloric acid, were measured for both ICS1 and ICS2 (Table 4). Naturally, the horse serum represents a wound-like environment as it is viscous, has a yellow color, and contains proteins. In the case of ICS1, the calculated pH values correlate well with the pH of horse serum measured by the pH electrode, while in the case of ICS2 the calculated values are slightly too high, presumably due to the intrinsic coloration of the horse serum, which may affect the a* value. Although both swabs show color changes from green to red, the spectra of both dyes are significantly different with different absorbance maxima for the base form (GJM-492: 492 nm, GJM-503: 503 nm), causing differences in the effect of the yellow color [43]. Eventually, a compensation measurement for the color of the real analyte sample (wound fluid) has to be devised for practical applications.

## 4. Conclusions

The pH-sensing system can give an indication of the progress of wound healing because wounds do not heal properly when at a pH above 8. We have successfully colored cotton swabs with the indicator and inert dye to possibly achieve two functions, namely (a) cleaning of the wound from its exudate and (b) simultaneous pH determination. Thus, the swabs may give valuable information on the healing process and can act as an early indicator for possible pathogenic processes. The next step will involve characterization of the indicator cotton swabs in a clinical study to establish proper measurement and calibration procedures for practical application by the clinical personnel, as well as evaluation of the correlation between pH and healing progress.

## Figures and Tables

**Table 1:** pKa values of the indicator cotton swabs before and after gamma sterilization.

	ICS1 (Not Sterilized)	ICS1 (Sterilized)	ICS2 (Not Sterilized)	ICS2 (Sterilized)
Five swabs measured once	5.89 (0.07)	5.85 (0.06)	7.38 (0.11)	7.34 (0.05)
One swab measured five times	5.75 (0.11)	5.87 (0.07)	7.38 (0.09)	7.37 (0.03)

**Table 2:** Effect of temperature on the pK_a_ value of the dyes in the indicator cotton swabs ICS1 and ICS2.

	ICS1 (n = 10)	ICS2 (n = 10)
pK_a_ at 20 °C	5.86 (0.22)	7.31 (0.26)
pK_a_ at 30 °C	5.92 (0.14)	7.39 (0.31)
pK_a_ at 40 °C	5.90 (0.08)	7.43 (0.13)

**Table 3:** Measurement of pH in phosphate buffer and Ringer solution before and after soaking wound dressings with them.

pH of Solutions Measured by a pH Electrode	Mepilex	AQUACEL Extra	Suprasorb A
Phosphate buffer (pH 6.0)	6.6	6.5	6.3
Phosphate buffer (pH 7.0)	7.3	7.5	6.6
Phosphate buffer (pH 8.0)	8.2	7.9	6.5
Ringer solution (pH 6.0)	7.1	5.1	5.2
Ringer solution (pH 7.0)	7.5	5.0	5.4
Ringer solution (pH 8.0)	7.7	5.1	5.6

**Table 4:** Measurement of pH in horse serum using ICS1 and ICS2.

pH of Horse Serum Measured by a pH Electrode	ICS1 *	ICS2 *
6.01	6.00 (0.06)	6.39 (0.04)
6.62	6.62 (0.18)	6.81 (0.02)
8.41	8.29 (0.36)	8.54 (0.30)

* Average of three measurements, standard deviation in brackets.
